# A rare mimic of adult‐onset asthma: Case report and review of the literature

**DOI:** 10.1002/rcr2.1225

**Published:** 2023-09-27

**Authors:** Jane Sandra Afriyie‐Mensah, Hafisatu Gbadamosi, Afua Owusua Darkwah Abrahams

**Affiliations:** ^1^ Department of Medicine and Therapeutics University of Ghana Medical School Accra Ghana; ^2^ Department of Radiology Korle Bu Teaching Hospital Accra Ghana; ^3^ Department of Radiology University of Ghana Medical School Accra Ghana; ^4^ Department of Pathology University of Ghana Medical School Accra Ghana

**Keywords:** adenoid cystic carcinoma, adult‐onset asthma, bronchoscopy, computed tomography, tracheal tumour

## Abstract

Adult‐onset asthma is extremely variable in its phenotypic presentation and has gained notoriety for the overall poorer treatment outcomes even on standard asthma therapy. Tracheal tumours are rare but when present, exhibit asthma‐like phenomenon in adult patients posing great diagnostic challenges. We report two adult patients with tracheal adenoid cystic tumours who were initially treated for adult‐onset asthma. Patient 1, a 45‐year‐old man was diagnosed and managed for adult‐onset asthma over a 12 months period without satisfactory control of his symptoms. Following a late episode of hemoptysis, a chest Computed Tomography (CT) scan done revealed an occluding tracheal tumour. Patient 2 is a 28‐year‐old female who was diagnosed with adult‐onset asthma for over 2 years with poor symptom control despite optimal asthma therapy. She developed cough‐induced subcutaneous emphysema for which a chest CT scan revealed a tracheal mass. The patient had surgery with incomplete resection of tumour and adjuvant radiotherapy.

## Graphical abstract

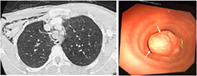

## INTRODUCTION

Diagnosis of primary tracheal tumours are infrequently made worldwide and represents approximately 0.2%–0.6% of all primary lung tumours.^1^ Its presentation can be non‐specific and insidious but mostly include signs and symptoms of upper airway obstruction, strongly mimicking common obstructive airway disorders such as asthma or COPD.^2^ Diagnosing tracheal tumours can therefore be difficult and mostly delayed among adults due to the presumptive diagnosis of asthma leading to poor outcomes.^2^, ^3^ The prevalence of tracheal tumours is lacking in Ghana with no case documentation among adults, probably due to missed diagnoses or limited availability of diagnostic resources. Highlighting these cases will help create awareness among clinicians particularly those who encounter patients with obstructive airway diseases.

## CASE REPORT

### Case 1

**FIGURE 1:**
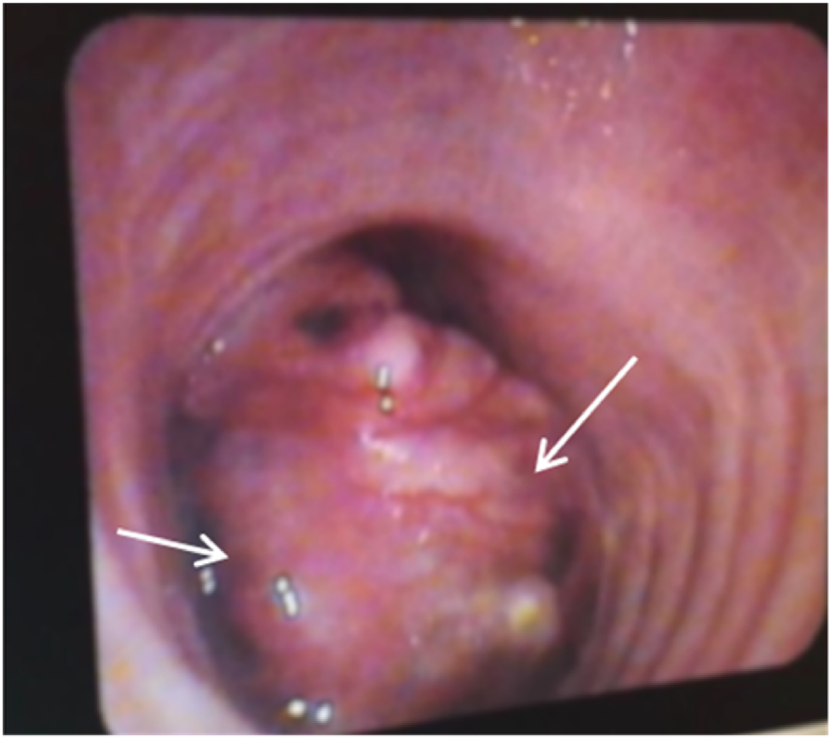
Image on bronchoscopy showing a partly occlusive lobulated mass in the distal trachea (white arrows).

**FIGURE 2:**
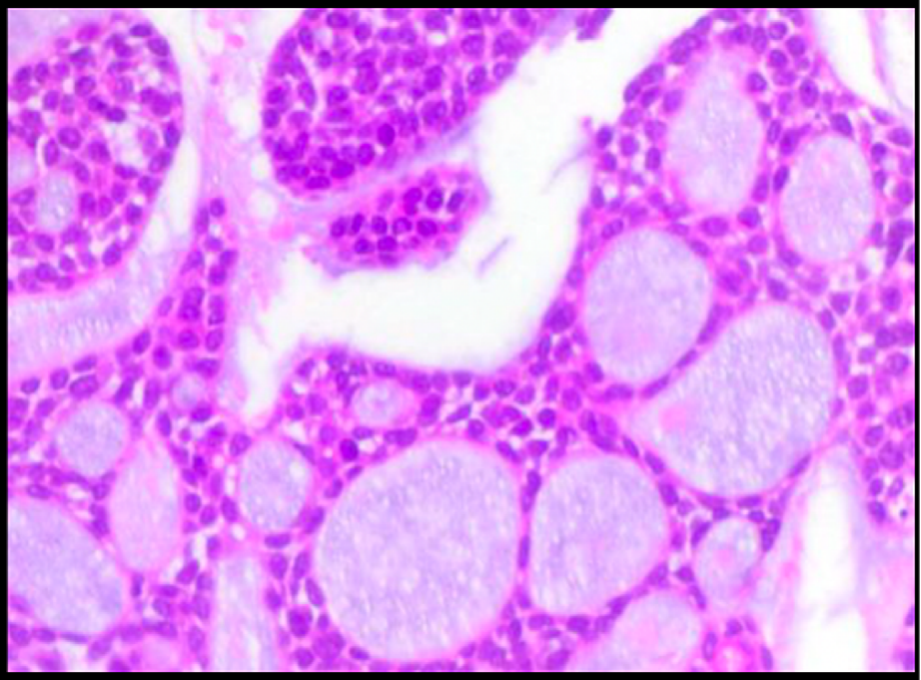
Photomicrograph (H and E stain ×10) Microscopy showing infiltrating tumour composed of uniform, small cuboidal cells in tubular /cribriform and solid patterns with small basophilic nuclei. Cystic spaces are filled with pale blue, mucoid material.

A 45‐year‐old man had been diagnosed with asthma about 12 months prior to presentation on account of recurrent episodes of breathlessness, central chest pain and cough productive of moderate volume clear phlegm. He had no personal or family history of asthma or allergies. There was no known triggers or diurnal variation of his symptoms. Initial therapy with salbutamol inhaler provided partial relief of acute symptoms and he was later placed on maintenance formoterol/budesonide inhaler. Persistence of his symptoms led to excessive use of salbutamol inhaler and repeated oral steroids with no long‐term improvement. Ten months into his symptoms, the patient noticed specks of blood in his sputum, this recurred with approximately 20–30mLs of coughed‐up blood. Consequently, a chest CT‐Scan ordered revealed a focal lesion in the trachea with normal lung parenchyma and no lymphadenopathy. Flexible bronchoscopy revealed a broad‐based tracheal tumour with a nodular surface located in the lower half of the trachea obstructing more than two‐thirds of the lumen (Figure 1). Biopsies confirmed tracheal adenoid cystic carcinoma, ACC (Figure 2). The tumour base was very broad hence the initial intention by the thoracic surgeon to snare and cauterize at bronchoscopy was not feasible. The patient underwent invasive surgery about 15 months after his initial symptoms through a posterolateral thoracotomy and pleural cavity entered the fourth intercostal space and was intubated using an appropriately sized ET tube. At thoracotomy, there was an obvious mass in the mediastinum arising from the trachea with no apparent lymph nodes involvement. There was a complete ring between the distal end of tumour and the carina and proximally, the tumour reached up to the mid‐trachea. With the ribs retracted, the lower portion of the trachea was in excellent view. The mediastinal pleural was entered and peritracheal tissues dissected free. The oesophagus was clearly displaced posteriorly, and membranous trachea easily separated from it. Once the trachea was circumferentially dissected, the distal end was opened and an armoured tube was placed in the left main bronchus (endobronchial intubation). The proximal end of the tumour was then identified and resected. With the specimen totally resected out of the way, 4.0 Polydioxanone PDS sutures were placed in the trachea rings starting from the left side, working sequentially to the anterior aspect of the trachea and then to the right and the cartilaginous rings of the two ends of the trachea were re‐approximated with no excessive tension on the anastomosis. The membranous trachea was subsequently closed with a running 4‐0 PDS suture. After the wound was irrigated, drains were placed, incision closed and the patient was extubated post‐operatively prior to being sent to the surgical ICU.

**FIGURE 3:**
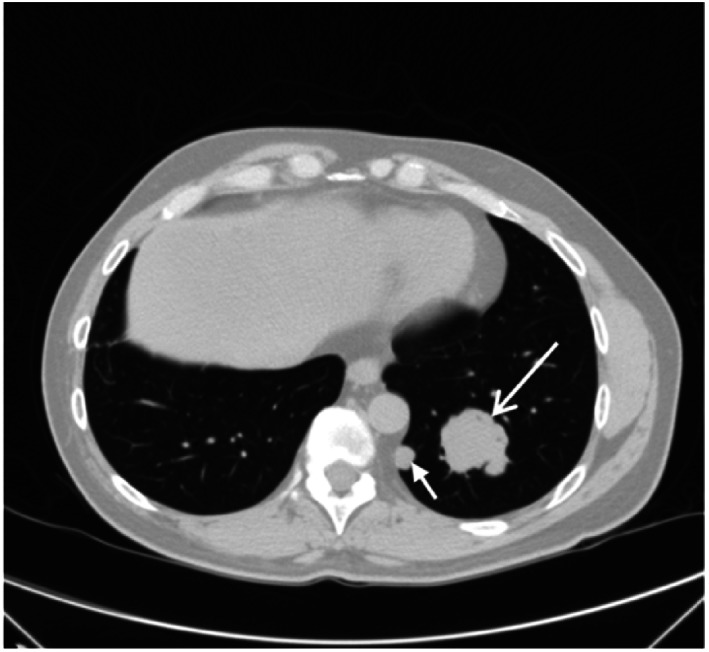
Axial CT scan of the chest in lung window showing a lobulated left lower lobe mass (long arrow) and a smaller adjacent pleural nodule (short arrow) indicative of metastases.

Post‐surgery, symptoms completely resolved. However, routine chest‐CT 6‐years post‐surgery showed bilateral enhancing lung nodules (Figure 3) histologically confirmed to be metastasis from ACC. Patient subsequently had a left lower lobectomy and radiofrequency ablation of the right lung mass in 2022. He is currently well with no recurrence on chest imaging.

### Case 2

**FIGURE 4:**
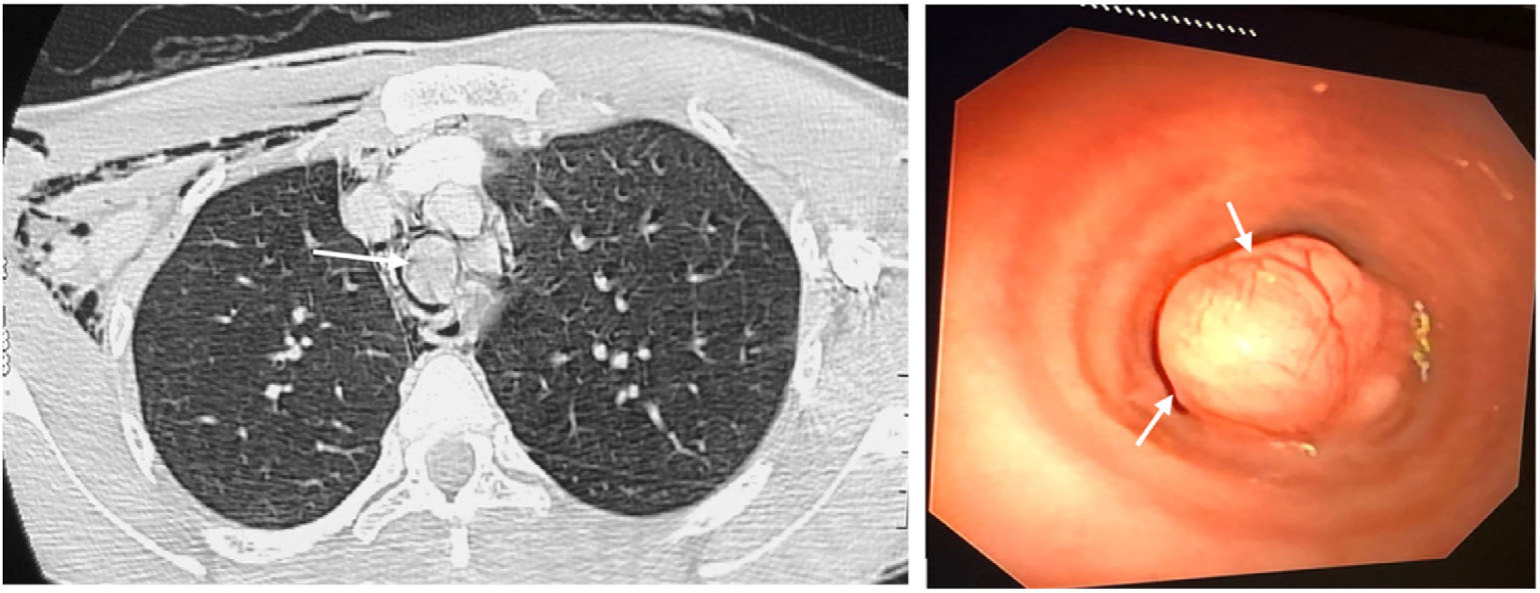
(A) Chest CT scan lung window image depicting the occluding tracheal mass (*long white arrow*) and right chest wall/ axillary soft tissue emphysema. (B) Bronchoscopy image demonstrating a smooth surfaced tracheal mass (*short white arrow*) causing severe airway stenosis.

A 28‐year‐old female with a childhood history of allergic rhinitis but no personal or family history of asthma experienced a sudden onset of unprovoked violent coughing with difficulty in breathing while at an event in August 2016. She had to be admitted and managed as acute bronchospasm secondary to an upper respiratory tract infection with nebulized salbutamol providing partial relief of her symptoms. After this incident, she experienced a gradual worsening of her otherwise tolerable rhinitis and recurrent episodes of cough, chest pain and shortness of breath with no relief from salbutamol inhaler. She was diagnosed with adult‐onset asthma and prescribed antihistamines, nasal steroids and formoterol/budesonide inhaler as maintenance treatment. Although the rhinitis improved, as‐needed salbutamol and optimal doses of maintenance inhaler did not prevent multiple hospital admissions due to acute asthma exacerbations. One occurrence of the violent coughing spells led to swelling around the neck and upper chest. This was diagnosed as subcutaneous emphysema on a chest CT‐scan requested which also revealed a mass in the proximal part of the trachea, shown (Figure 4A). Biopsy at bronchoscopy confirmed tracheal adenoid cystic carcinoma 28 months after initial presentation (Figure 4B). At surgery, the pleural cavity was entered via the 4th intercostal space and Superior Vena Cava displaced anteriorly. Though the trachea was identified, the tumour was not visible or palpable but the proximal and distal ends were identified using the bronchoscope. The tumour was 5 cm below the vocal cords and attached anteriorly to the trachea. The trachea was opened distally and resected. The trachea was intubated through the distal incision while the trachea at the proximal end of the tumour was incised and transected. An extra tracheal ring was excised proximally as the tumour was very close to the line of excision. With the tumour completely removed, the anastomosis was performed using a 3/0 and 4/0 PDS sutures. Once the anastomosis was partially complete, an oral endotracheal tube was advanced past the anastomosis for ventilation and subsequent completion of the end‐to end anastomosis.

Pathology demonstrated macroscopic findings of portions of the trachea measuring 18 × 22 × 22 mm with a large polypoid tumour of about 19 × 15 × 16 mm which was about 2 mm from the closest margin. Evidence of tracheal cartilage infiltration was present.

**FIGURE 5:**
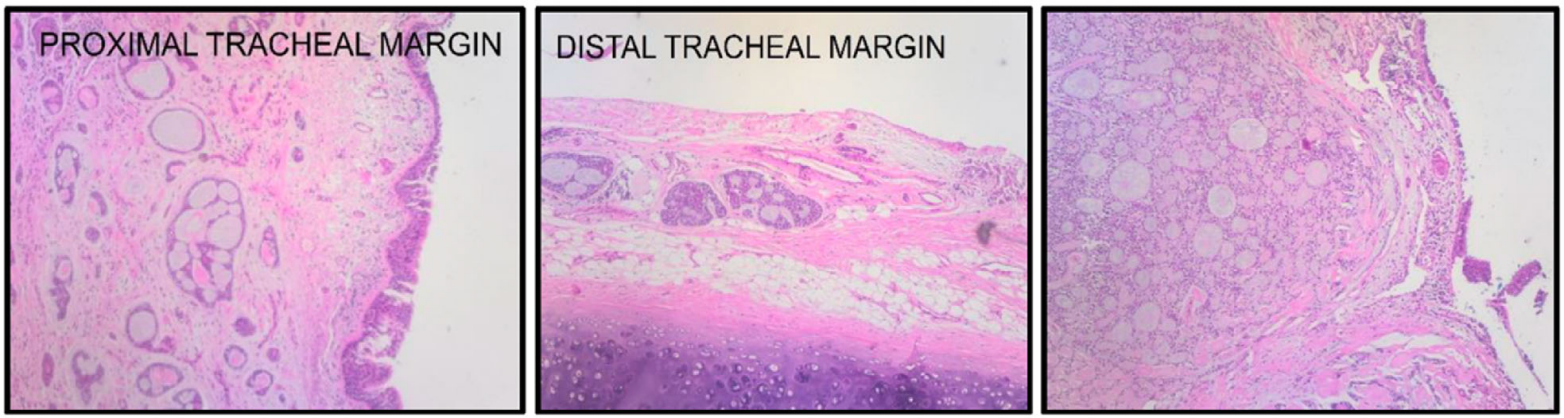
(H&E ×10) Microscopic images showing cribriform structures with abundant basement membrane like material and evidence of submucosal involvement and tracheal cartilage involvement.

Microscopic images of sections of the tumour confirm the presence of a large tumour representive of a classic adenoid cystic carcinoma. The tumour showed cribriform structures with abundant basement membrane like material with evidence of perineural infiltration as shown in Figure 5.

Post‐operatively, she underwent adjuvant radiation therapy due to the positive tissue margins on histology. Patient remains well 4 years onwards with no asthma‐like symptoms ever experienced except infrequent symptoms of allergic rhinitis. Follow‐up chest CT scan have showed no lung or local tumour recurrence.

## DISCUSSION

Primary tracheal tumours in adults tend to be malignant with squamous cell carcinoma (SCC) and adenoid cystic carcinoma (ACC) accounting for 45% and 16.3%, respectively.^4^ The cause of tracheal ACC is unknown whiles smoking is a major risk factor for tracheal SCC.^5^ This was corroborated in both cases which were non‐smokers, with no other identifiable risk factors. Tracheal ACC typically presents in the 4th and 5th decade of life. While case 1 fits this age profile case 2 was much younger at age 28. A review by Högerle et al however reported a wider age range of 19–80 years.^6^

The clinical presentation of tracheal tumours is variable with an insidious course explained by their slow growing nature and the trachea's propensity to accommodate the tumour size till about 50% of its lumen is obstructed.^7^ Compared with tracheal SCC, tracheal ACC exhibits a longer interval of 18 months between onset of symptoms and diagnosis due their much slower growth.^6^, ^8^ Our cases also had long intervals of 12 and 28 months respectively. Tracheal tumours commonly present with signs and symptoms of airway obstruction such as dyspnea, cough, and wheezing which explains the misdiagnosis as adult‐onset asthma in the majority of cases.^2^ Dyspnea and cough as reported by our patients appear to be the commonest symptoms observed in 86% and 58% of patients with tracheal tumours.^8^ Other symptoms such as hemoptysis, dysphagia and hoarseness are usually late symptoms stemming from invasion of the overlying mucosa, adjacent oesophagus and recurrent laryngeal nerve respectively.^8^ Adult‐onset asthma is defined as onset of asthma symptoms for the first time in adulthood, typically at or above 18 years.^9^ Its global prevalence is unknown but estimated to be about 4.5%, although variable among different populations.^10^ Epidemiological studies have shown that a good proportion of patients with adult‐onset asthma, tend to have a severe asthma phenotype with increased risk of exacerbations and poorer disease outcomes.^11^ This clinical picture unfortunately tends to cloud the thought of other differential diagnoses in such patients who exhibit frequent asthma exacerbations, shifting the focus to treatment optimization as happened in our patients. It is therefore recommended that a thorough history and examination is carried out especially in patients with adult‐onset asthma to exclude differential diagnoses. Symptoms and examination findings suggestive of other diagnoses include stridor often confused as wheeze, hemoptysis, weight loss, and persistent/uncontrolled symptoms despite optimal therapy or presence of a localized wheeze on chest auscultation.^12^

Chest radiograph has low specificity and sensitivity for detecting tracheal tumours, being diagnostic in just about 18%–28% of cases.^13^ Careful visualization of the trachea and the central airways should be performed on all patients presenting with symptoms of stridor, wheezing and adult‐onset asthma.^13^ The investigation of choice is thus computed tomography (CT) and Magnetic Resonance imaging (MRI), which clinched the diagnosis in our cases.^13^ It is worth noting, that chest CT is typically not a required diagnostic investigation in asthma, however, its role among patients with severe asthma symptoms is being highlighted in recent times, as the persistent so‐called “asthma symptoms” could arise from diverse differential diagnoses such as endobronchial tumours, interstitial pneumonias, bronchiectasis, associated COPD and bronchiolitis.^14^ Even when asthma diagnosis is secured, associated complications and comorbidities such as allergic bronchopulmonary aspergillosis, eosinophilic pneumonitis and bronchiectasis which are diagnosed on chest CT scans, could be making the background asthma appear severe and difficult to control.^14^ In our cases, the persistence of the patients' “asthma‐like” symptoms in spite of optimal treatment should have prompted early referral and request for a chest CT scan much earlier than the onset of alarming symptoms. This could have avoided the undue diagnostic delays.

In the absence of advanced disease, surgical resection of tracheal tumours is the mainstay of treatment.^15^ Although there are no clear‐cut recommendations, detection of positive tissue margins on histology is one of the reasons for adjuvant radiotherapy as tracheal ACC tumours are highly radiosensitive.^6^, ^8^ In line with the above recommendation, adjuvant radiotherapy was withheld in case 1, whiles case 2 had adjuvant radiotherapy. Irrespective of surgical margins, combined surgical and adjuvant radiotherapy treatment modality is preferred as complete tumour resection is not absolutely achieved in many patients.^16^ Survival rates for surgery alone is 86% and 55.6% at 5 and 10 year follow‐up respectively, whiles combination therapy had higher rates at 97% and 44.4%, respectively.^8^ Late lung metastases is common with tracheal ACC, hence the need for follow‐up bronchoscopy and imaging.^6^ This practice led to the discovery of bilateral pulmonary metastasis in case 1 in the current report.

In conclusion, tracheal tumours by their nonspecific symptoms have a protracted clinical course and are mostly misdiagnosed as adult‐onset asthma. To improve survival, a high index of suspicion is required, especially among patients labelled as adult‐onset asthma with poor disease control despite optimal therapy and early chest CT‐scan requested to include or exclude other differential diagnoses among adults.

## AUTHOR CONTRIBUTIONS

All authors contributed to preparation of the manuscript and approve the content.

## CONFLICT OF INTEREST STATEMENT

None declared.

## ETHICS STATEMENT

The authors declare that appropriate written informed consents were obtained for the publication of this manuscript and accompanying images.

## Data Availability

Data sharing is not applicable to this article as no new data were created or analyzed in this study.

## References

[1] Junker K . Pathology of tracheal tumors. Thorac Surg Clin. 2014;24(1):7–11. 24295655 10.1016/j.thorsurg.2013.09.008

[2] Rees CJ , Pollack CV Jr , Blanck JF . Tracheal tumors. In: Pollack C , editor. Differential diagnosis of cardiopulmonary disease. Cham: Springer; 2019.

[3] Napieralska A , Miszczyk L , Blamek S . Tracheal cancer: treatment results, prognostic factors and incidence of other neoplasms. Radiol Oncol. 2016;50(4):409–417. 27904449 10.1515/raon-2016-0046 PMC5120581

[4] Madariaga MLL , Gaissert HA . Overview of malignant tracheal tumors. Ann Cardiothorac Surg. 2018;7(2):244–254. 29707502 10.21037/acs.2018.03.04 PMC5900094

[5] Yang P‐Y , Liu M‐S , Chen C‐H , Lin C‐M , Tsao TCY . Adenoid cystic carcinoma of the trachea: a report of seven cases and literature review. Chang Gung Med J. 2005;28(5):357–363. 16086551

[6] Högerle BA , Lasitschka F , Muley T , Bougatf N , Herfarth K , Adeberg S , et al. Primary adenoid cystic carcinoma of the trachea: clinical outcome of 38 patients after interdisciplinary treatment in a single institution. Radiat Oncol. 2019;14(1):117. 31272473 10.1186/s13014-019-1323-z PMC6610895

[7] Brand‐Saberi BEM , Schäfer T . Trachea: anatomy and physiology. Thorac Surg Clin. 2014;24(1):1–5. 24295654 10.1016/j.thorsurg.2013.09.004

[8] Ran J , Qu G , Chen X , Zhao D . Clinical features, treatment and outcomes in patients with tracheal adenoid cystic carcinoma: a systematic literature review. Radiat Oncol [Internet]. 2021;16(1):38. 33608038 10.1186/s13014-021-01770-0 PMC7893857

[9] de Nijs SB , Venekamp LN , Bel EH . Adult‐onset asthma: is it really different? Eur Respir Rev. 2013;22(127):44–52. 10.1183/09059180.00007112 Erratum in: Eur Respir Rev 2013 Jun 1;22(128):193. 23457164 PMC9487439

[10] Teresa T , Stanojevic S , Moores G , Gershon AS , Bateman ED , Cruz AA , et al. Global asthma prevalence in adults: findings from the cross‐sectional world health survey. BMC Public Health [Internet]. 2012;12(1):204. 10.1186/1471-2458-12-204 22429515 PMC3353191

[11] Ilmarinen P , Juboori H , Tuomisto LE , Niemelä O , Sintonen H , Kankaanranta H . Effect of asthma control on general health‐related quality of life in patients diagnosed with adult‐onset asthma. Sci Rep. 2019;9(1):16107. 31695074 10.1038/s41598-019-52361-9 PMC6834611

[12] Lalloo UG , Kalla IS , Abdool‐Gaffar S , Dheda K , Koegelenberg CFN , Greenblatt M , et al. Guidelines for the management of asthma in adults and adolescents: position statement of the South African Thoracic Society: 2021 update. African J Thorac Crit Care Med. 2021;27(4):187–199. 10.7196/AJTCCM.2021.v27i4.189 PMC8802209 35118373

[13] Shepard J‐AO , Flores EJ , Abbott GF . Imaging of the trachea. Ann Cardiothorac Surg. 2018;7(2):197–209. 29707497 10.21037/acs.2018.03.09 PMC5900079

[14] Ash SY , Diaz AA . The role of imaging in the assessment of severe asthma. Curr Opin Pulm Med. 2017;23(1):97–102. 27775932 10.1097/MCP.0000000000000341 PMC5244861

[15] Kumar NS , Iype EM , Thomas S , Sankar UV . Adenoid cystic carcinoma of the trachea. Indian J Surg Oncol. 2016;7(1):62–66. 27065684 10.1007/s13193-015-0453-5 PMC4811809

[16] Levy A , Omeiri A , Fadel E , Le Pechoux C . Radiotherapy for tracheal‐bronchial cystic adenoid carcinomas. Clin Oncol (R Coll Radiol). 2018;30:39–4618. 29122457 10.1016/j.clon.2017.10.012

